# Complete chloroplast genome structural characterization of two *Aerides* (Orchidaceae) species with a focus on phylogenetic position of *Aerides flabellata*

**DOI:** 10.1186/s12864-024-10458-0

**Published:** 2024-06-03

**Authors:** Kaifeng Tao, Lei Tao, Jialin Huang, Hanning Duan, Yan Luo, Lu Li

**Affiliations:** 1 College of Forestry, Southwest Forestry University, Kunming, Yunnan 650224 China; 2 School of Chemistry, Biology and Environment, Yuxi Normal University, Yuxi, Yunnan 653100 China; 3 Southeast Asia Biodiversity Research Institute, Chinese Academy of Sciences & Center for Integrative Conservation, Xishuangbanna Tropical Botanical Garden, Chinese Academy of Sciences, Mengla, Yunnan China

**Keywords:** *Aerides flabellata*, *Aerides rosea*, Chloroplast genome, Orchidaceae, *Vanda*-*Aerides* alliance, Structural characterization

## Abstract

**Background:**

The disputed phylogenetic position of *Aerides flabellata* Rolfe ex Downie, due to morphological overlaps with related species, was investigated based on evidence of complete chloroplast (cp) genomes. The structural characterization of complete cp genomes of *A. flabellata* and *A. rosea* Lodd. ex Lindl. & Paxton were analyzed and compared with those of six related species in “*Vanda*-*Aerides* alliance” to provide genomic information on taxonomy and phylogeny.

**Results:**

The cp genomes of *A. flabellata* and *A. rosea* exhibited conserved quadripartite structures, 148,145 bp and 147,925 bp in length, with similar GC content (36.7 ~ 36.8%). Gene annotations revealed 110 single-copy genes, 18 duplicated in inverted regions, and ten with introns. Comparative analysis across related species confirmed stable sequence identity and higher variation in single-copy regions. However, there are notable differences in the IR regions between two *Aerides* Lour. species and the other six related species. The phylogenetic analysis based on CDS from complete cp genomes indicated that *Aerides* species except *A. flabellata* formed a monophyletic clade nested in the subtribe Aeridinae, being a sister group to *Renanthera* Lour., consistent with previous studies. Meanwhile, a separate clade consisted of *A. flabellata* and six *Vanda* R. Br. species was formed, as a sister taxon to *Holcoglossum* Schltr.

**Conclusions:**

This research was the first report on the complete cp genomes of *A. flabellata*. The results provided insights into understanding of plastome evolution and phylogenetic relationships of *Aerides*. The phylogenetic analysis based on complete cp genomes showed that *A. flabellata* should be placed in *Vanda* rather than in *Aerides*.

**Supplementary Information:**

The online version contains supplementary material available at 10.1186/s12864-024-10458-0.

## Background

*Aerides* Lour. (Aeridinae, Vandeae, Epidendroideae, Orchidaceae) consists of about 29 species, which are distributed from India to Papua New Guinea [1–3]. There are five species recorded in China, including one endemic species, which occurs in Southern China [4]. The distinct fragrance emitted by *Aerides* species has made them a valuable source for the production of numerous artificial hybrids and cultivars [5].

*Aerides* has been a focus of taxonomic disagreement within the subtribe Aeridinae [3, 5–7]. Since *Aerides* was first described, many members previously placed in other genera have been moved into it [7]. Conversely, dozens of species once included in *Aerides* have now been removed into other related genera [7]. The intrageneric taxonomy of *Aerides* were questioned due to the transfer of several species to other genera, such as *Ornithochilus* (Lindl.) Wall. ex Heynh., *Papilionanthe* Schltr., and *Seidenfadenia* Garay [8, 9]. *Aerides* was characterized by the presence of two cleft pollinia and divided into five groups based predominantly on pollinia morphology [10, 11]. However, two cleft pollinia were observed in other related genera, including *Brachypeza* Garay, *Phalaenopsis* Bl., *Rhynchostylis* Bl., *Vanda* R. Br. and among others [7]. Then, the concept of the “*Vanda*-*Aerides* alliance”, comprising *Aerides*, *Ascocentrum* Schltr., *Holcoglossum* Schltr., *Neofinetia* Hu, *Papilionanthe*, *Rhynchostylis* and *Vanda*, was proposed [12], while the intergeneric delimitation has been controversial based on nuclear DNA data [3]. It is worth mentioning that the phylogenetic position of *Aerides flabellate* Rolfe ex Downie has been a focus issue [13, 14]. It was placed in *Aerides* based on an analysis using a plastid *matK* gene [15], but moved into *Vanda* in the latter treatment supported by an analysis of combined DNA datasets (nrITS and *matK*, *trnL*, *trnL-F*) [16].

The chloroplast (cp) genome has been increasingly utilized in taxonomy and phylogeny of Orchidaceae [17–19]. The complete cp genomes of six *Aerides* species (*Aerides crassifolia* C. S. P. Parish ex Burb., *Aerides falcata* Lindl. & Paxton, *Aerides lawrenceae* Rchb.f., *Aerides odorata* Lour., *Aerides quinquevulnera* Lindl., and *Aerides rosea* Lodd. ex Lindl. & Paxton) were published [20]. The results indicated that *Aerides* should be a separate clade within Aeridinae, sister to *Renanthera* Lour [20]. However, it should be noted that the complete cp genomic data of *A. flabellata* have not been reported. In this study, the structural and genomic information of the cp genomes of *A. flabellata* and *A. rosea* was characterized in detail and compared with those of six related species in the “*Vanda*-*Aerides* alliance”. The objectives of this study were: (1) to characterize and compare the complete cp genome structures of *A. flabellata* and *A. rosea* in detail, (2) to reconstruct the phylogenetic tree of Aeridinae to verify the position of *A. flabellata*, and (3) to provide new genomic data for a better understanding of the phylogeny of *Aerides*.

## Results

### General data on the chloroplast genome

**Fig. 1:**
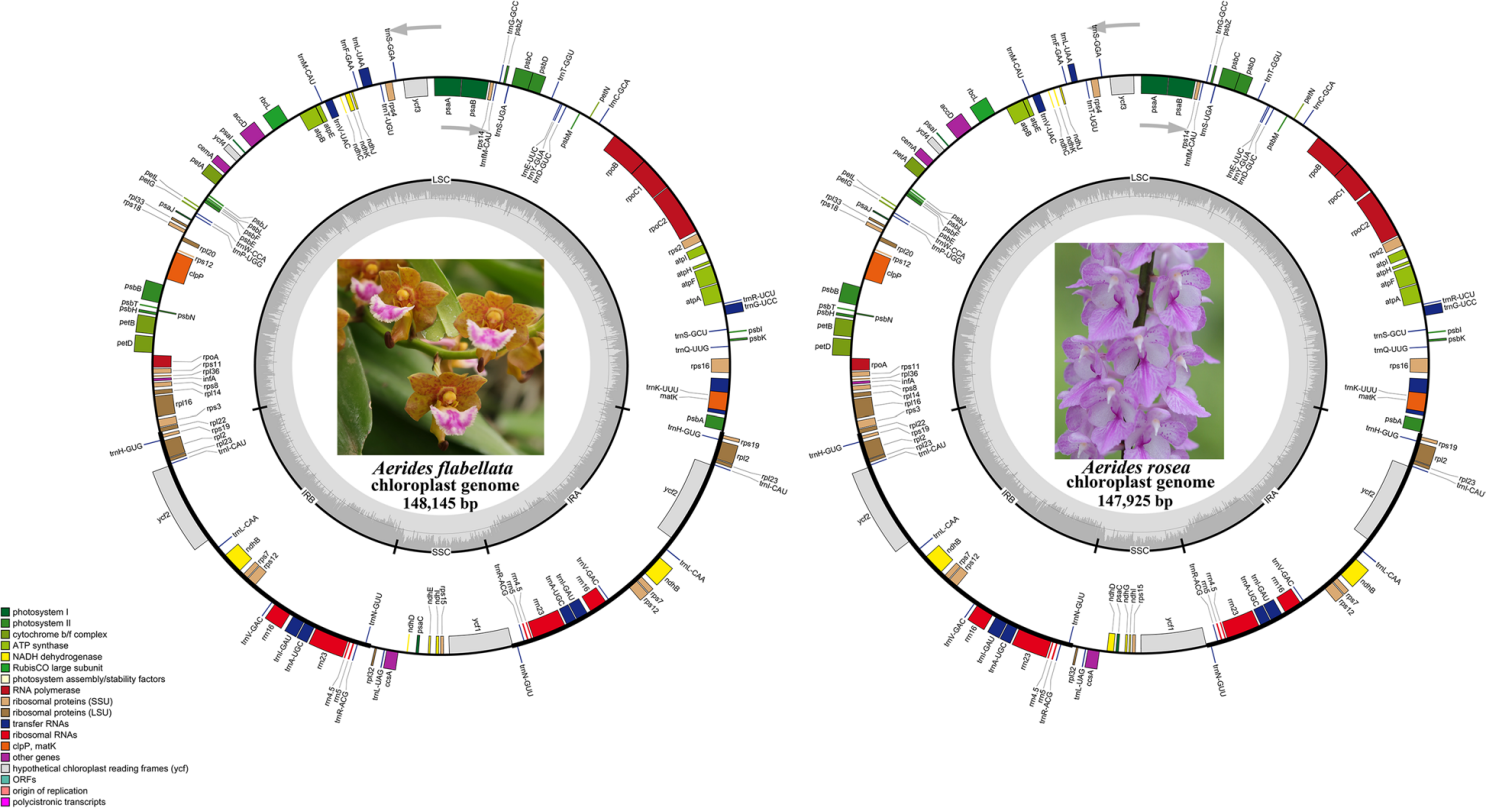
The chloroplast genome maps of *Aerides flabellata* and *A. rosea*. Internal genes were clockwise transcribed, while external genes were counterclockwise transcribed. The inside circle bright and dark gray coloring indicated the genome guanine-cytosine (GC) content

The depth of the assemblies was 494.99 (*Aerides flabellata*) and 240.80 (*A. rosea*) (Fig.S1). The structures of cp genomes of the two *Aerides* species are highly similar. The total sizes of two cp genomes were 148,145 bp (*A. flabellata*) and 147,925 bp (*A. rosea*) (Fig. 1, Table 1). Same as most angiosperms, their cp genome displayed a typical quadripartite structure with a large single-copy (LSC) region (84,905 bp, 85,317 bp), a small single-copy (SSC) region (11,636 bp, 11,018 bp), and two inverted repeats (IR) regions (25,802 bp, 25,795 bp). The two cp genomes were all AT-rich, overall GC content ranged from 36.7 ~ 36.8%. The GC content in IR regions (43.1 ~ 43.2%) was higher than in LSC (34 ~ 34.1%) and SSC regions (28.82%) (Table 1). The GC content of the three codon positions of the two cp genomes was very similar. Furthermore, the third codon position was related to codon bias and mRNA stability. However, the third letter GC (36.28%) content was lower than the first (37.18%) and second (36.80%) letter GC content in *A. flabellata*. In contrast, the third letter GC content (36.53%) was lower than the second (37.18%) letter GC content, but higher than the first letter GC (36.49%) content in *A. rosea* (Table 2). Both cp genomes contained 128 genes, including 2 (*A. flabellata*) ~ 3 (*A. rosea*) pseudogenes, 79 (*A. rosea*) ~ 80 (*A. flabellata*) CDS (coding sequences), eight rRNAs, and 38 tRNAs (Table 1). Among these, there were 110 unique genes in each cp genome. The LSC region contained 62 CDS genes and 21 tRNA genes in the two cp genomes. The SSC region comprised only one tRNA gene in the two cp genomes but eight CDS genes in *A. flabellata* and seven CDS genes in *A. rosea*. Six CDS genes (*rpl2*, *rpl23*, *rps7*, *rps12*, *rps19*, and *ycf2*), eight tRNA genes (*trnA-UGC*, *trnH-GUG*, *trnI-CAU*, *trnI-GAU*, *trnL-CAA*, *trnN-GUU*, *trnR-ACG*, and *trnV-GAC*), and four rRNA genes (*rrn4.5*, *rrn5*, *rrn16*, and *rrn23*) were repeated in the IR regions (Table S1). There were ten genes with introns in the two cp genomes, seven genes with one intron (*rps16*, *rpoC1*, *rpl2*, *rpl16*, *petD*, *petB*, and *atpF*), and the other three genes with two introns (*clpP*, *ycf3*, *rps12*) (Table S2). However, the length of ten intron-containing genes were different in the two *Aerides* species (Table S2). Only one of the ten intron-containing genes were in the IR regions, while the other genes spread across the LSC region. In addition, *rps12* was a unique trans-splicing gene in which the first exon dispersed in the LSC region, but the second and third exons were in IR regions. Seven *ndh* (NA (D)H dehydrogenase) genes were identified in the cp genome of *A. flabellata* (*ndh B*/*C*/*D*/*E*/*I*/*J*/*K*) and *A. rosea* (*ndh B*/*C*/*D*/*G*/*I*/*J*/*K*) (Fig. 1, Table S1).

**Table 1:** The general genome characteristics of the two *Aerides* species

Characteristics and Parameters	*Aerides flabellata*	*Aerides rosea*
Total cp genome size (bp)	148,145	147,925
LSC length (bp)	84,905	85,317
SSC length (bp)	11,636	11,018
IR length (bp)	25,802	25,795
Total GC content (%)	36.8	36.7
GC content for LSC (%)	34.1	34
GC content for SSC (%)	28.2	28.2
GC content for IR (%)	43.1	43.2
Total number of genes	128	128
CDS genes	80	79
rRNAs genes	8	8
tRNAs genes	38	38
Pseudogenes	2	3

**Table 2:** The GC content of the three positions of the two *Aerides* species

Species	1st letter GC	2nd letter GC	3rd letter GC
*Aerides flabellata*	37.18%	36.80%	36.28%
*A. rosea*	36.49%	37.23%	36.53%

### Repeat sequences analysis

The number of SSRs was analyzed to elucidate allied species or intra-species variations. There were 57 (*Aerides flabellata*) and 76 (*A. rosea*) SSRs detected in the two cp genomes, respectively consisting of 39 mononucleotides, seven dinucleotides, four trinucleotides, five tetranucleotides, one pentanucleotide and one hexanucleotide in *A. flabellata,* but of 52 mononucleotides, 12 dinucleotides, six trinucleotides, four tetranucleotides, two pentanucleotides in *A. rosea* (Table 3). Repeat units were composed mainly of A or T, and the mononucleotides were A/T type rather than G/C type in the two cp genomes. Furthermore, the C/G mononucleotide and AAAT/ATTT type tetranucleotide only existed in *A. flabellata* (Fig. S2).

**Table 3:** The number of SSRs types distributed in different copy regions of the two *Aerides* species

**SSRs types**	***Aerides flabellata***	***Aerides rosea***
Mononucleotide	39	52
Dinucleotide	7	12
Trinucleotide	4	6
Tetranucleotide	5	4
Pentanucleotide	1	2
Hexanucleotide	1	0
Total	57	76

Four different types of long repeats were also identified based on the complete genome sequence: complement (C), forward (F), palindromic (P), and reverse (R) (Table S3). Forty-nine large repeats were detected in the two cp genomes. In *A. flabellata*, almost all the repeats ranged from 20 to 39 bp, with the fewest in 40 ~ 49 bp. However, the number of long repeats above 40 bp in length was similar to the repeats from 20 to 39 bp in *A. rosea*. No complement repeats were detected above 40 bp in length, and they were rare even in the smaller size ranges (Table S3).

### Codon usage analysis

Based on coding sequences (CDS), codon usage frequency and relative synonymous codon usage (RSCU) were computed in the cp genomes of the two *Aerides* species and other six related species from “*Vanda*-*Aerides* alliance” (*Aerides falcata*, *A. lawrenceae*, *A. odorata*, *Vanda coerulea* Griff. ex Lindl., *V. coerulescens* Griff., and *V. subconcolor* Tang & F. T. Wang) downloaded from NCBI (https://www.ncbi.nlm.nih.gov) (Table S4) [21]. These CDS were composed of 48,830 to 49,803 codons, respectively, and encoded 20 amino acids in the eight cp genomes (Fig. S3, Table S4). The RSCU value of seven chloroplast genomes was similar, except *A. odorata*, which possessed the lower RSCU of leucine (Leu) and the higher RSCU of serine (Ser). Among them, leucine (Leu: 9.65 ~ 10.46%) was the amino acid that was utilized the most frequently, whereas tryptophan (Trp: 1.27 ~ 1.45%) was the least ubiquitous amino acid in the eight cp genomes (Table S5). According to the RSCU value, the eight cp genome could be divided into five groups: 28 codons (RSCU > 1) and 33 codons (RSCU < 1) in *A. odorata*; 29 codons (RSCU > 1) and 31 codons (RSCU < 1) in *A. falcata*; 30 codons (RSCU > 1) and 32 codons (RSCU < 1) in *A. flabellata* & *V. coerulea*; 31 codons (RSCU > 1) and 30 codons (RSCU < 1) in *A. lawrenceae* & *V. subconcolor*; 31 codons (RSCU > 1) and 31 codons (RSCU < 1) in *V. coerulescens*; 32 codons (RSCU > 1) and 30 codons (RSCU < 1) in *A. rosea* (Table S4). Almost all CDS in the eight species had the standard ATG start codon, but *rpl2* started with ATA/TAT. Among three stop codons, the TAA was the most common.

### IR expansion and contraction

**Fig. 2:**
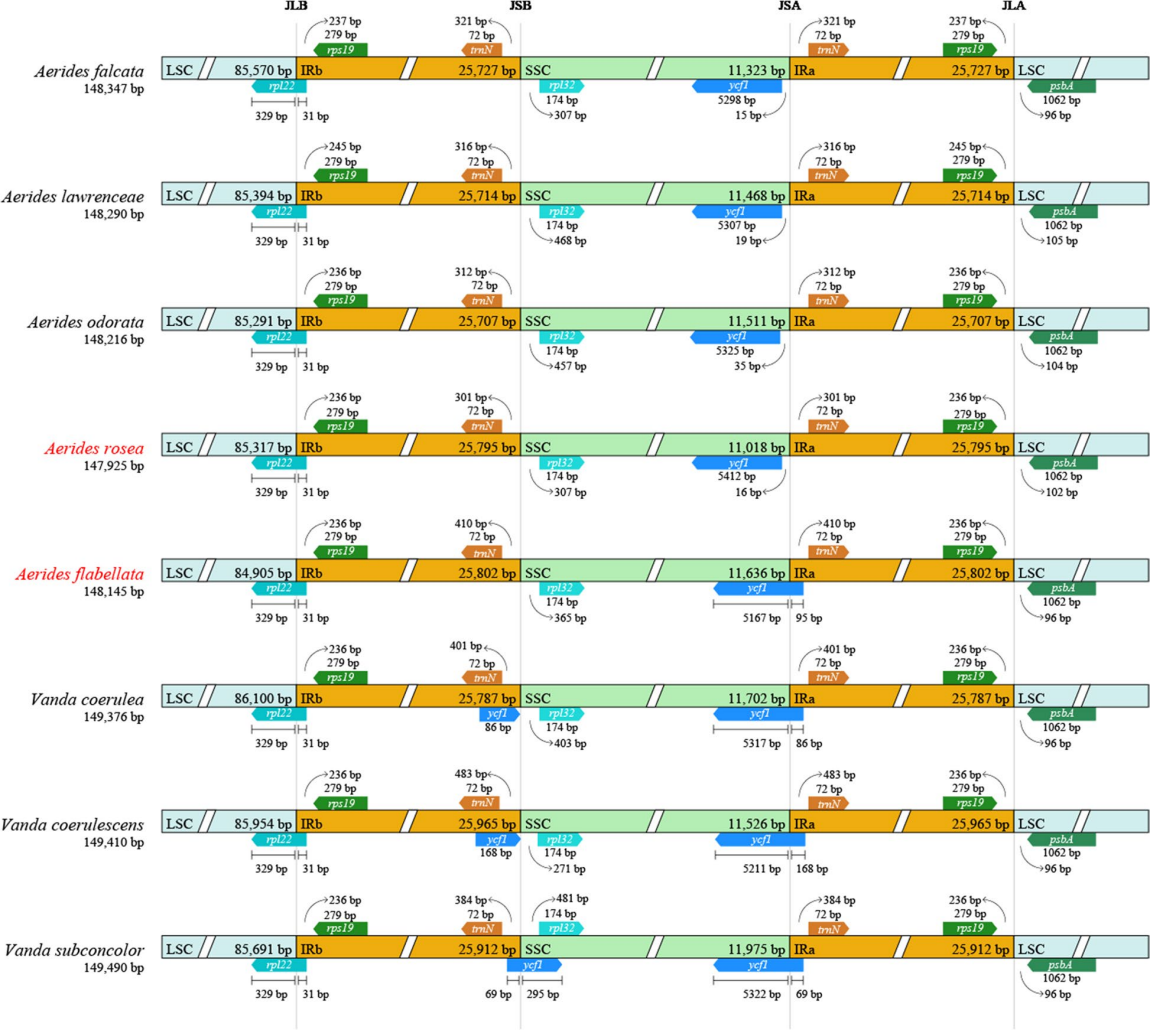
Comparison of the boundaries of LSC, SSC and IR regions among chloroplast genomes of the two *Aerides* species and six species selected from “*Vanda-Aerides* alliance”. The arrow indicated the number of bp representing genes that were distant from a particular region of the cp genome. JLB (LSC/IRb), JSB (IRb/SSC), JSA (SSC/IRa), and JLA (IRa/LSC) denoted the junction sites between each corresponding two regions on the cp genome

The cp genomes of the two *Aerides* species were highly conserved structurally, as well as those of the six species selected from “*Vanda-Aerides* alliance”. There were four boundaries (LSC/IRb, IRb/SSC, SSC/IRa, IRa/LSC) with structural variations (Fig. 2). The *rpl22* gene was expanded from LSC to the IRb region. The *rpl32* gene was present in the SSC region in the eight species. The *trnN* gene was observed in the IRa and IRb region in the eight species. Notably, the *ycf1* gene was expanded from SSC to the IRa region in *A. flabellata* and three *Vanda* species, while it was only located in the SSC region in the other four *Aerides* species. In addition, the *ycf1* gene was also present in the IRb region of *V. coerulea* and *V. coerulescens,* and it expanded from IRb to the SSC region in *V. subconcolor*, but it is absent in *A. flabellata* and *A. rosea.*

### Structural comparison and divergence hotspot identification analysis

**Fig. 3:**
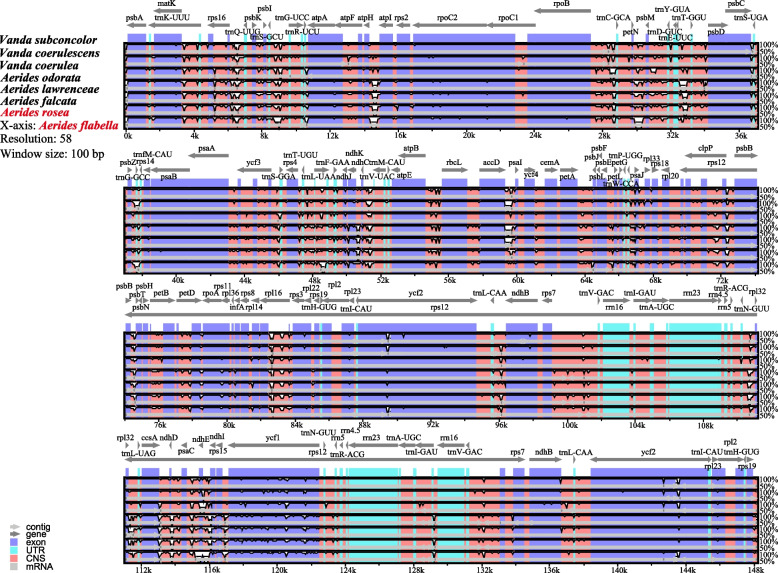
Sequence alignment of chloroplast genomes of the two *Aerides* species and six species selected from “*Vanda-Aerides* alliance” using mVISTA. The vertical scale indicates the percentage of identity, ranging from 50 to 100%. The horizontal axis indicated the coordinates within the cp genome. Genome regions were color coded as exon, intron, and conserved non-coding sequences (CNS) and mRNA

Using *Aerides flabellata* as the reference, the cp genome sequences were compared by mVISTA (Fig. 3). The IR regions were more stable than the LSC and the SSC regions, and the rRNA genes were highly conserved. Meanwhile, the non-coding regions (CNS) were more diverse than the coding regions. The exons of *ycf1* and *ycf2* gene exhibited the highest polymorphism.

**Fig. 4:**
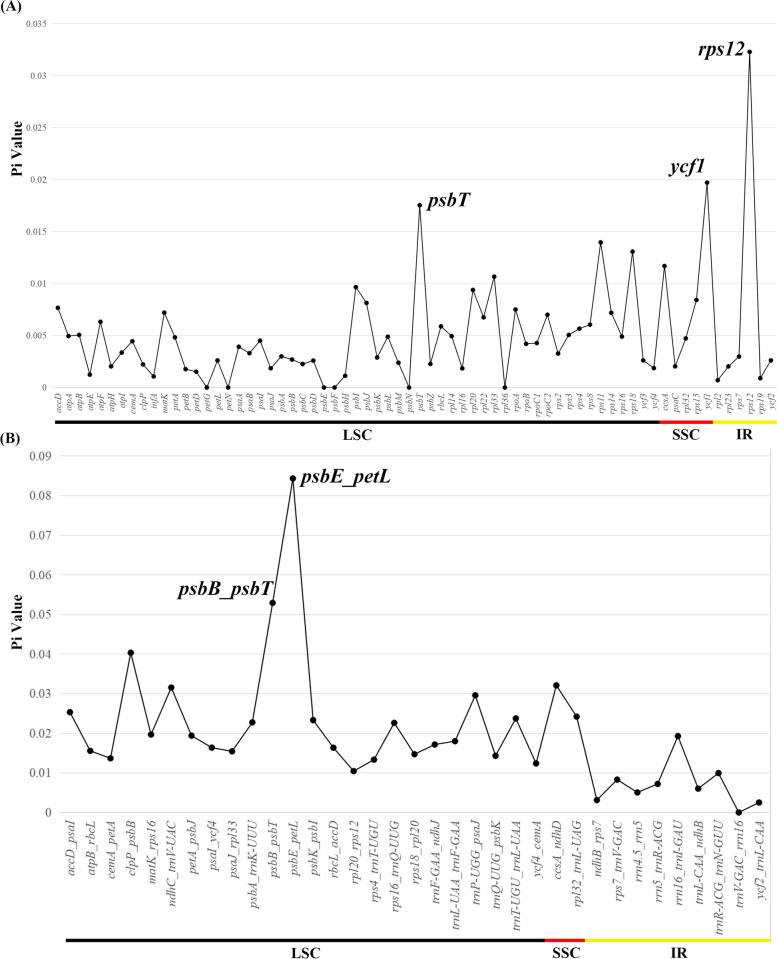
Sliding window analysis of cp genomes of two *Aerides* species and six species selected from “*Vanda-Aerides* alliance”. **A** Comparison of the nucleotide diversity (Pi) among CDS regions. **B** Comparison of the nucleotide diversity among IGS regions. X-axis: position of the midpoint of a window; Y-axis: nucleotide diversity of each window. Highest variation hotspots for eight cp genomes are annotated on the graph. The colored lines at the bottom delineate these gene locations in different regions

It was shown that the Pi value of LSC and SSC regions was greater than those of the IR regions based on the examination of CDS DNA polymorphism, demonstrating that the former were more varied than the latter. Three out of 62 CDS possessed the highest Pi values: *psbT* (0.01753), *ycf1* (0.01970) and *rps12* (0.03228) (Fig. 4A, Table S5). There were two locations with high Pi value (> 0.05) for the IGS (intergenic spacer), including *psbB_psbT* (0.05291) and *psbE_petL* (0.08433) (Fig. 4B, Table S6). The Pi value of IGS locations (0.00 ~ 0.07, average 0.01965) was greater than that of CDS (0.00 ~ 0.024, average 0.00505) (Fig. 4, Table S5, S6).

### Positive selection analysis

The Bayes Empirical Bayes (BEB) method identified 53 genes under positive selection, with *rpl22*, *rps4*, *rps8*, *rps14*, *rps16*, *rps18*, *rpl32*, *ycf1*, and *ycf2* genes having two or more significant positive selection sites. Other genes had just one substantial positive selection site aside. The number of positive selections of genes in LSC was higher than in SSC and IR regions (Table 4, Table S7).

**Table 4:** The positive selection analysis of two *Aerides* species and six species selected from “*Vanda-Aerides* alliance”

M8							
Region	Gene name	Positive sites	Pr(ω > 1)	Region	Gene name	Positive sites	Pr(ω > 1)
LSC	*atpA*	508	0.988*	LSC	*rpl22*	55 K	0.982*
LSC	*atpE*	135	1.000**	LSC		58 Y	0.982*
LSC	*atpH*	82	1.000**	LSC		87 I	0.982*
LSC	*atpI*	248	1.000**	LSC		88 V	0.982*
LSC	*cemA*	230	1.000**	LSC		110 N	0.982*
LSC	*infA*	78	1.000**	LSC		120	1.000**
LSC	*petA*	321	1.000**	LSC	*rps4*	166 I	0.965*
LSC	*petB*	216	1.000**	LSC		202	1.000**
LSC	*petD*	164	1.000**	LSC	*rps8*	3 R	0.967*
LSC	*petG*	38	1.000**	LSC		132	1.000**
LSC	*petL*	32	1.000**	LSC	*rps11*	139	1.000**
LSC	*petN*	30	1.000**	LSC	*rps14*	22 F	0.960*
LSC	*psaB*	735	0.991**	LSC		101	1.000**
LSC	*psaI*	37	1.000**	LSC	*rps16*	34 Q	0.977*
LSC	*psaJ*	45	1.000**	LSC		38 F	0.977*
LSC	*psbA*	354	1.000**	LSC		86 K	0.977*
LSC	*psbB*	509	1.000**	LSC	*rps18*	4 F	0.964*
LSC	*psbD*	354	1.000**	LSC		102	1.000**
LSC	*psbE*	84	1.000**	LSC	*ycf4*	185	1.000**
LSC	*psbF*	40	1.000**	SSC	*ccsA*	322	1.000**
LSC	*psbH*	74	1.000**	SSC	*psaC*	82	1.000**
LSC	*psbI*	37	1.000**	SSC	*rpl32*	52 K	0.957*
LSC	*psbJ*	41	1.000**	SSC		58	1.000**
LSC	*psbK*	62	1.000**	SSC	*ycf1*	829 E	0.971*
LSC	*psbL*	39	0.999**	SSC		1020 L	0.976*
LSC	*psbM*	35	1.000**	IR	*rpl2*	272	1.000**
LSC	*psbN*	44	1.000**	IR	*rpl23*	94	1.000**
LSC	*psbT*	36	1.000**	IR	*rps7*	156	1.000**
LSC	*psbZ*	63	1.000**	IR	*rps19*	93	1.000**
LSC	*rpl14*	123	1.000**	IR	*ycf2*	562 S	0.994**
LSC	*rpl16*	136	1.000**	IR		563 G	0.985*
LSC	*rpl20*	118	1.000**	IR		564 C	0.989*
LSC	*rpl33*	67	1.000**	IR		771 M	0.971*
LSC	*rpl36*	38	1.000**	IR		1562 D	0.972*
LSC	*rpoA*	338	1.000**	IR		1782 N	0.969*
LSC	*rps3*	219	1.000**				

*p > 95%; **p > 99%

### Phylogenetic analysis

**Fig. 5:**
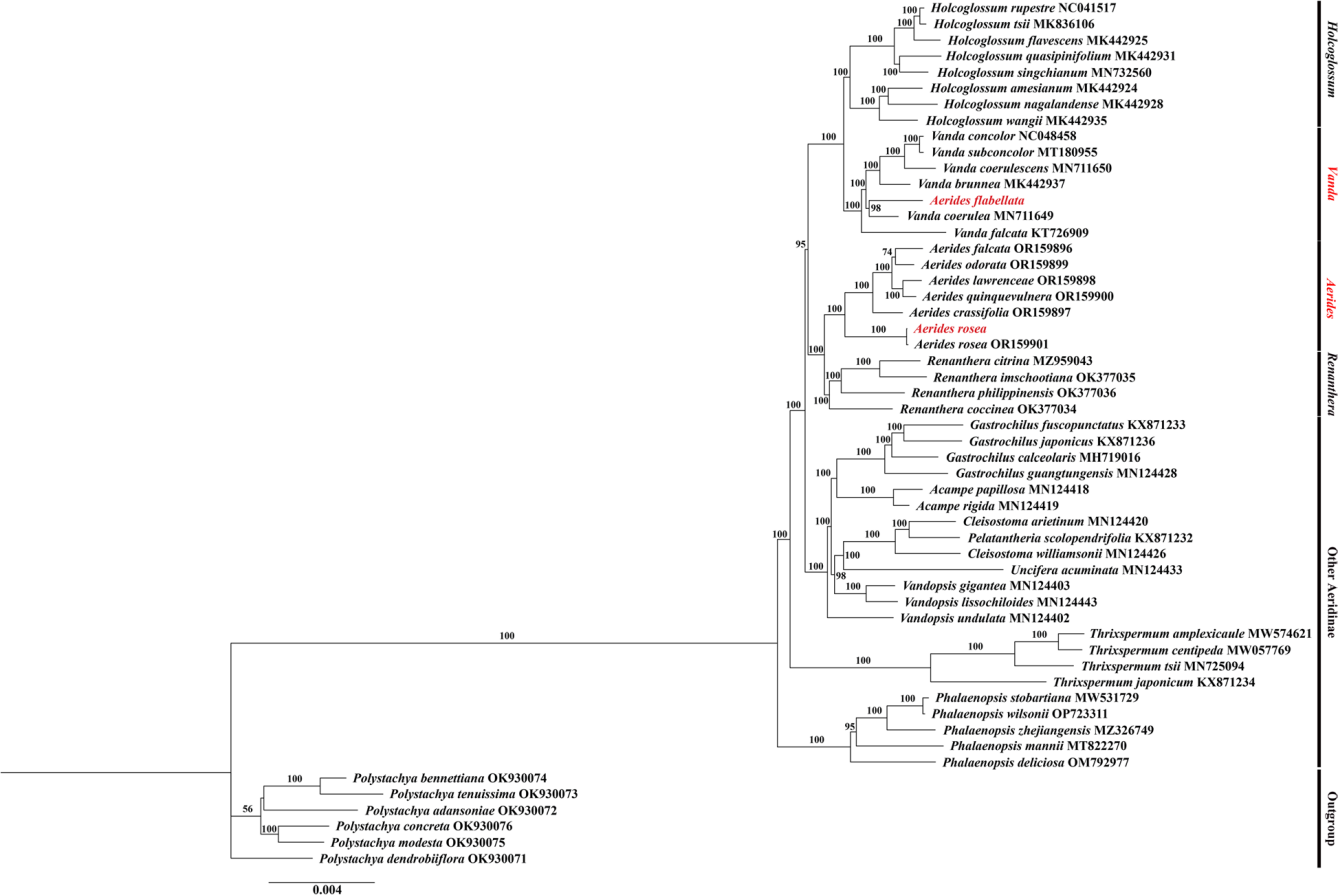
Phylogenetic tree reconstructed of Aeridinae using Maximum-likelihood (ML) method based on 62 single-copy CDS sequences of 47 Aeridinae species, with six *Polystachya* species as outgroups

A Maximum-likelihood (ML) phylogenetic tree was reconstructed based on 62 single-copy CDS sequences of the two *Aerides* species and 45 representatives from Aeridinae, with six *Polystachya* species as outgroups, to shed a light on the phylogeny of *Aerides*, as well as the position of *A. flabellate* (Fig. 5, Table S8). *A. flabellata* and six *Vanda* species were formed as a stable clade with strong support (UFBoot: 100%), which was sister to *Holcoglossum* in the “*Vanda-Aerides* alliance”. It was shown that *A. flabellata* should be placed in *Vanda*, which was sister to *V. coerulea* with strong support (UFBoot: 98%). Meanwhile, six *Aerides* species formed a monophyletic clade, with *A. rosea* as the sister taxon to the other five species. This monophyletic clade of *Aerides* was also found to be sister to *Renanthera*. All the branch nodes in the clade of *Aerides* were strongly supported by the ML analysis.

## Discussion

In this study, the complete cp genomes of *Aerides flabellata* and *A. rosea* were sequenced and compared with those of other six related species within “*Vanda*-*Aerides* alliance” to learn more about the cp genomic information and the molecular phylogeny of *Aerides*.

The cp genomes of *Aerides flabellata* and *A. rosea* were highly similar. Both cp genomes showed a typical quadripartite circular structure with the LSC and SSC regions partitioned by the IR regions, which were similar to the other orchids and most of the angiosperms with no significant differences [19, 22]. Notably, the genome size differed from previous research, with 79 ~ 80 CDS were annotated in these two cp genomes, as opposed to the 74 CDS reported previously [20]. The annotation of the *ndh* CDS caused this difference. *A. flabellata* and *A. rosea* contained seven *ndh* genes with five ~ six *ndh* CDS. In contrast, other *Aerides* species lacked some *ndh* genes or *ndh* CDS [20]. Eleven *ndh* genes in cp genomes encode the NAD(p)H dehydrogenase [23]. Previous research delineated Apostasioideae as *ndh*-complete, Vanilloideae as *ndh*-deleted, Cypripedioideae, Orchidoideae, and Epidendroideae as both *ndh*-complete and *ndh*-deleted. These findings suggested the presence of a complete functioning set of *ndh* genes in the common ancestor of orchids [24]. In certain photoautotrophic plants, the NDH complex is deemed unnecessary [24, 25]. Additionally, the GC content of the IR regions was much higher than that of the LSC and SSC regions, and these characteristics were also observed in *Cardamine* species [26]. This phenomenon is caused by the presence of rRNA and tRNA genes in the IR regions, which is the same as in other Orchidaceae cp genomes [18, 19].

Simple sequence repeats (SSRs), also known as microsatellites, represent shorter tandem repeats consisting of 1 ~ 6 bp repeat units dispersed widely across the cp genome, and could be used for phylogenetic analysis [18, 27–29]. A total of 57 SSRs were identified in *Aerides flabellata*, while 76 were detected in *A. rosea*. Notably, the count of SSRs in *A. flabellata* diverged from recent research on *Aerides*, which reported a total of 71 ~ 77 SSRs [20]. Mononucleotide repeats emerged as the most prevalent SSRs within the cp genomes of both *A. flabellata* and *A. rosea*. Similar to six *Polystachya* species and three *Bulbophyllum* species, cp SSRs are predominantly comprised of short poly-A or poly-T repeats, and the mononucleotide repeats are the most commonly encountered forms [18, 30]. Repeated sequences play a pivotal role in species evolution, as well as in the inheritance and variation of genes within species [31, 32]. These repetitive sequences were widely used in the studies on genetic diversity, population structure, and the identification of closely related species [20, 33, 34]. In this study, 49 long repeats were identified from the two *Aerides* cp genomes, indicating that the *Aerides* cp genome retained abundant genetic information. The above findings can provide a data basis for further studies on population genetics.

The formation of codons is a critical process in translating genetic information from mRNA to protein [35], which is influenced by codon bias, particularly the third base usage pattern [36]. It has been empirically established that the GC composition exerts an influence on the utilization of codons and amino acids, and the GC content of the third codon base (GC3) is deemed to most closely reflect codon usage trends [37]. Regarding *Aerides* species, the GC content observed in this study aligns with previous research [20]. Based on the RSCU analysis, six codons encoded arginine, leucine and serine. However, only one codon encoded methionine and tryptophan, which was also reported in other orchid species [19, 38].

The IR region is the most conservative section within the cp genome. However, its boundaries have demonstrated frequent contractions and expansions, associated with the evolution of the cp genome, representing the primary driver for variations in cp genome length [39, 40]. Unlike basal angiosperms and eudicots, most monocots typically harbor *trnH-rps19* clusters in each IR region [41]. In this study, the *trnH-rps19* clusters were also located in each IR region, which was consistent with other five *Aerides* species [20], *Paphiopedilum henryanum* Braem [42], *Phalaenopsis stobartiana* Rchb.f., *P. wilsonii* Rolfe [19], and *Platanthera ussuriensis* (Regel) Maxim [17]. The presence of the *trnH-rps19* gene cluster in the IR of most monocots has been suggested as evidence of a duplication event predating the divergence of monocot lineages. Contractions and expansions in the IR borders have also been proposed to implicate taxonomic relationships among angiosperms [27, 41]. Additionally, *Aerides crassifolia*, *A. quinquevulnera*, *A. lawrenceae*, *A. odorata*, and *A. falcata* were consistent with *A. rosea* [20], wherein the *ycf1* gene was exclusively located in the SSC region. In contrast, the *ycf1* gene spanned the SSC and IRa regions in *A. flabellata*, aligning with observations in *Vanda subconcolor*.

Divergent regions, serving as valuable sources of data for DNA barcoding and phylogenetic research, were frequently employed as molecular markers in studies focused on phylogenetic reconstruction [43]. In this study, the nucleotide sequence of non-coding regions was more varied than the coding regions, which was generally consistent with other Orchidaceae cp genomes [18, 19]. Furthermore, the analysis of coding sequence regions revealed that the genes *rps12*, *psbT* and *ycf1* had significantly higher Pi values. Notably, *ycf1*, akin to *matK*, has been utilized as a DNA marker for phylogenetic studies [43]. In this research, *psbB_psbT* and *psbE*_*petL* also possessed the higher degree of variability. Simultaneously, sequences such as *trnS_trnG*, *psaC_ndhE*, *clpP_psbB*, and others exhibited the highest degree of variability in *Phalaenopsis* [19], while *rpl32_trnL*, *trnE_trnT*, and others showed the highest degree of variability in *Cymbidium* Sw. [44]. These indicated a diversity array of highly variable sequences in the Orchidaceae cp genome.

The utilization of the substitution rate ratio at synonymous and nonsynonymous sites (dN/dS, ω) has been pivotal in discerning adaptive signals among species and inferring evolutionary processes [45, 46]. Additionally, it could suggest that environmental factors impacted the evolution of cp genomes, representing a primary cause for the divergence of numerous genes within the cp genome [47]. In this study, 53 genes were significantly identified under positive selection. Among them, the *atpH*, *petL*, and *rps4* genes have also been observed in other orchids [19, 48]. Furthermore, these genes could be used for orchid identification and phylogenetic research.

*Aerides flabellata* (synonym: *Vanda flabellata*) has been a focus of considerable taxonomic disagreement [6, 49]. Some taxonomists placed it within *Aerides* on account of features such as a long column foot and motile lip [10], while others assigned it to *Vanda*, emphasizing the species’ short spur and broad lip [3, 5, 8, 50]. The species *Christensonia vietnamica* Haager, exhibiting morphological resemblances to both *Vanda* and *Rhynchostylis* [13], has been affiliated with *A. flabellata*, being described as ‘almost a yellow *Aerides flabellata*’ [13]. Therefore, *A. flabellata* and *C. vietnamica* were placed into *Vanda* based on combined DNA datasets (nrITS and *matK*, *trnL*, *trnL-F*) [3, 6, 15, 51].

The structural features of the cp genome have been utilized in constructing the phylogeny of Orchidaceae [17–19], because protein-coding regions and conserved sequences were informative for taxonomy [52]. In this study, based on CDS data from complete cp genomes, it was showed that *Aerides flabellata* was embedded within the clade of *Vanda*, while other six *Aerides* were grouped into a stable monophyletic clade. Therefore, it was supported that *A. flabellata* should be moved into *Vanda* from *Aerides* based on the comparative and the phylogenetic analyses.

## Conclusion

The complete cp genomes of *Aerides flabellata* and *A. rosea* were sequenced and analyzed to unveil their genomic intricacies. This investigation encompassed a holistic exploration of various facets, including the general genome structure, codon usage, repeat sequences, boundaries within the inverted repeats, DNA polymorphism, and phylogenetic position. These cp genomic datasets were compared with the other six related species from the “*Vanda*-*Aerides* alliance”. It was confirmed that the cp genomic features of the “*Vanda*-*Aerides* alliance” was almost congruent and highly conserved, which could be used to understand the plastome evolution and evolutionary relationships of the “*Vanda*-*Aerides* alliance”. In addition, it was supported that *A. flabellata* should be removed into *Vanda* from *Aerides* based on cp genomic data.

## Materials and methods

### Ethical statement

No specific permits were required for the collection of specimens for this study. This research was carried out in compliance with the relevant laws of China.

### Plant materials and chloroplast genome sequencing

Leaf samples of *Aerides flabellata* and *A. rosea* were cultivated and obtained from the Xishuangbanna Tropical Botanical Garden, Chinese Academy of Sciences, Yunnan. The specimen was deposited in the Herbarium of Southwest Forestry University (HSFU, Lilu20180015, lilu@swfu.edu.cn). Genomic DNA of each sample was extracted from the silica gel-dried leaf tissues using the modified CTAB method with the TiangenDNA kit (TIANGEN, China) [53]. Paired-end libraries with an average insert size of approximately 400 bp were prepared using a TruSeq DNA Sample Prep Kit (Illumina, Inc., San Diego, CA, USA) according to the manufacturer’s instructions. The libraries were sequenced on the Illumina HiSeq 2500 platform at Personalbio (two times 150 bp; Illumina, Shanghai, China). Raw data were filtered using Fastp v0.23.1 to obtain high-quality reads by the sliding window method to drop the low-quality bases of each read’s head and tail [54].

### Chloroplast genome assembly and annotation

The two complete cp genomes from the clean reads were assembled by the GetOrganelle version 1.7.7.0 [55] and annotated the new sequences using the Geneious Prime version 2020.0.4 [56]. The complete cp genomes sequences of *Aerides flabellata* and *A. rosea* were submitted to GenBank (Accession number: PP003956 and PP003955). The circular genome maps were drawn by the OGDRAW program (https://chlorobox.mpimp-golm.mpg.de/OGDraw) [44].

### Sequence analysis and statistics

The repetitive structures, repeat sizes, and locations of forward match (F), reverse match (R), palindromic match (P), and complementary match (C) nucleotide repeat sequences were identified by REPuter v2.74 (https://bibiserv.cebitec.uni-bielefeld.de/reputer/) [57], with maximal repeat size se to 50 bp, minimal repeat size set to 20 bp, and hamming distance set to 3 [20]. By setting the minimum number of repeats to 10, 5, 4, 3, and 3 for mononucleotide (mono-), dinucleotide (din-), trinucleotide (tri-), tetranucleotide (tetra-), pentanucleotide (penta-), and hexanucleotide (hexan-), respectively, simple sequence repeats (SSR), a tract of repetitive DNA that typically ranges in length from 1 to 6 nucleotides, were detected via MISA (https://webblast.ipk-gatersleben.de/misa/index.php?action=1) [58, 59]. Condon usage was analyzed by MEGA11 software [60], and the relative synonymous codon usage (RSCU) and amino acid frequencies were calculated with default settings [61]. Finally, the RSCU figure was drawn by PhyloSuite version 1.2.2 [62, 63]. In addition, the GC content of the three position was analyzed by CUSP on EMBOSS program (http://emboss.toulouse.inra.fr/cgi-bin/emboss/cusp) [64].

### Sequence divergence and genome comparison

The pairwise alignments and sequence divergence of *Aerides flabellata* and *A. rosea* with other six related species from “*Vanda-Aerides* alliance” (Table S9) were performed by the mVISTA with Shuffle-LAGAN mode (https://genome.lbl.gov/cgi-bin/VistaInput?num_seqs=2) [65]. Using an online application CPJSdraw v1.0.0 (http://112.86.217.82:9929/#/tool/alltool/detail/335), the contraction and extension of the IR borders between the four major areas (LSC/IRa/SSC/IRb) of the eight cp genome sequences were performed [66].

### Positive selection analysis

The CDS sequences of *Aerides flabellata* and *A. rosea* with other six related species from “*Vanda-Aerides* alliance” (Table S9) were extracted by PhyloSuite version 1.2.2 [62, 63], and the single-copy CDS sequences were aligned by MAFFT version 7 [67]. The phylogenetic tree based on CDS was platformed by MEGA 11 with Neighbor-Joining (NJ) methods [60]. The non-synonymous (dN) and synonymous (dS) substitution rates were calculated by the CodeML algorithm implemented in EasyCodeML [68] and selected the M8 mode for selection suites to detect the protein-coding genes under selection in the two *Aerides* species and six related species.

### Phylogenetic analysis

For phylogenetic analysis, the cp genomes of 53 species were selected (Table S9). The ingroup contains the genomes of 47 Aeridinae species, which 45 species were downloaded from the NCBI database. As Polystachyinae was sister to Aeridinae [18], six species from Polystachyinae were selected as outgroups. The single-CDS sequences (Table S8) from cp genomes were used for the phylogenetic analysis. These single-CDS sequences were extracted by PhyloSuite version 1.2.2 [62, 63], aligned by MAFFT version 7 [67], trimmed by Gblocks [69], and concatenated by plugins in PhyloSuite version 1.2.2 [62, 63]. The Maximum-Likelihood (ML) tree was performed in GTR + F + R2 mode based on CDS sequences by IQ-TREE 2 with 5000 ultrafast bootstrap (UFBoot) [70–72].

### Supplementary Information

Supplementary Material 1.

Supplementary Material 2.

Supplementary Material 3.

Supplementary Material 4.

## Data Availability

The datasets generated or analyzed during the current study are available in the NCBI BioProject (PRJNA994440 and PRJNA995179, SRA: SRR25256624 and SRR25293872).
