# Supplementary material for: Complete chloroplast genome structural characterization of two Aerides (Orchidaceae) species with a focus on phylogenetic position of Aerides flabellata
Source: BMC Genomics. 2024 Jun 3;25:552. doi: 10.1186/s12864-024-10458-0 (PMC11145882; doi:10.1186/s12864-024-10458-0)
Supplement: Supplementary file 1 — Supplementary Material 1. [file 12864_2024_10458_MOESM1_ESM.doc]

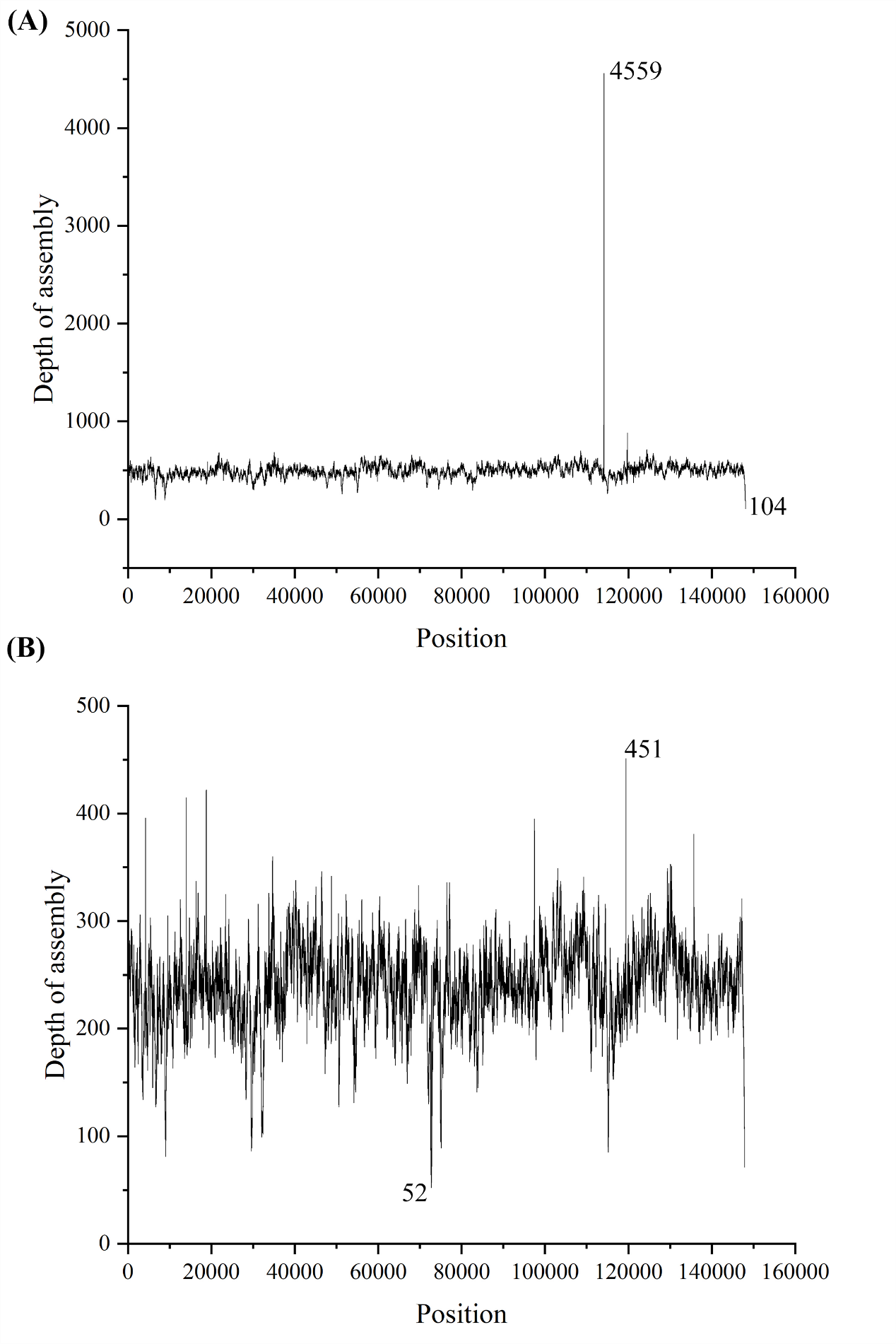


Fig. S1 The depth of assemblies about *Aerides flabellate* (A) and *A. rosea* (B).


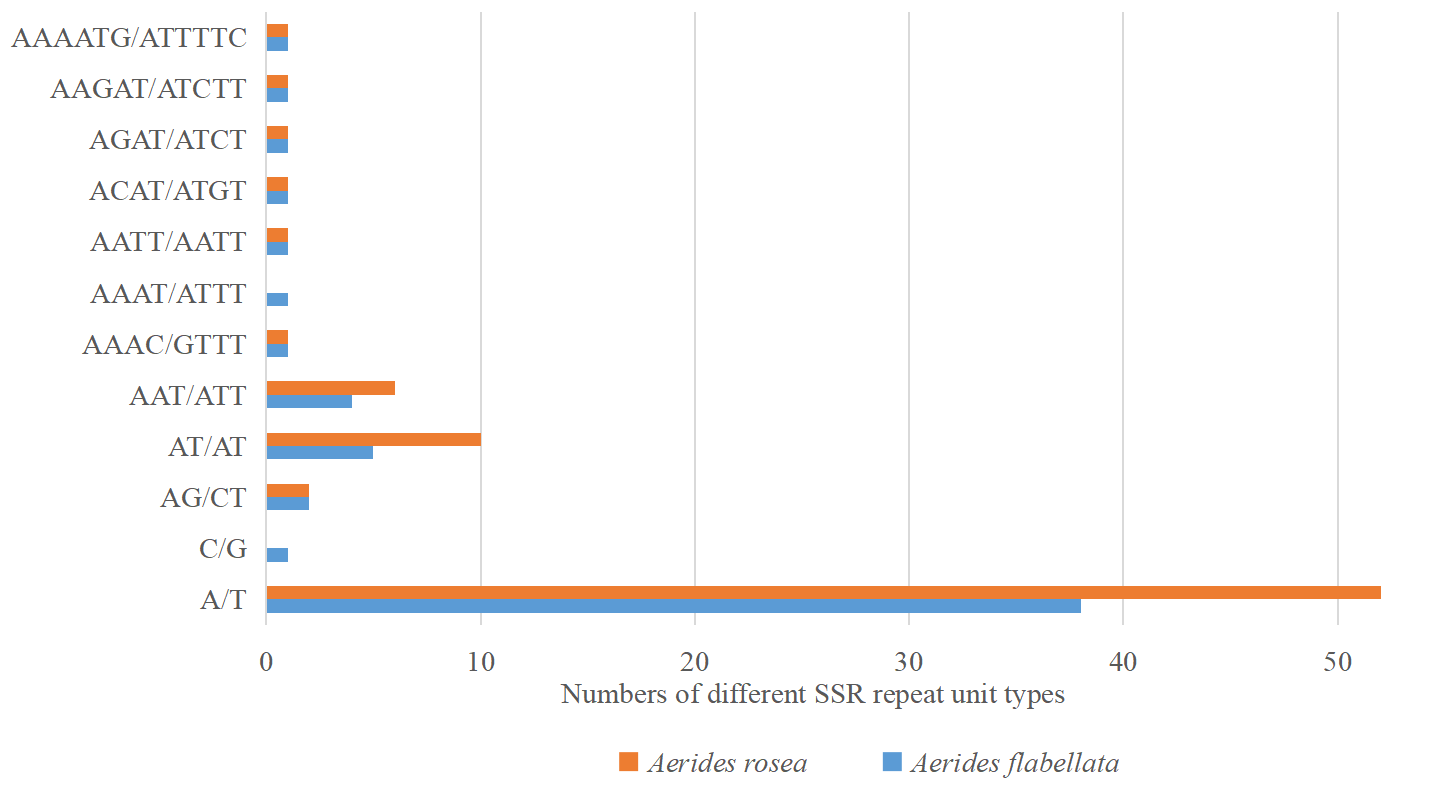


Fig. S2 The number of different SSR repeat unit types of the two *Aerides* species.


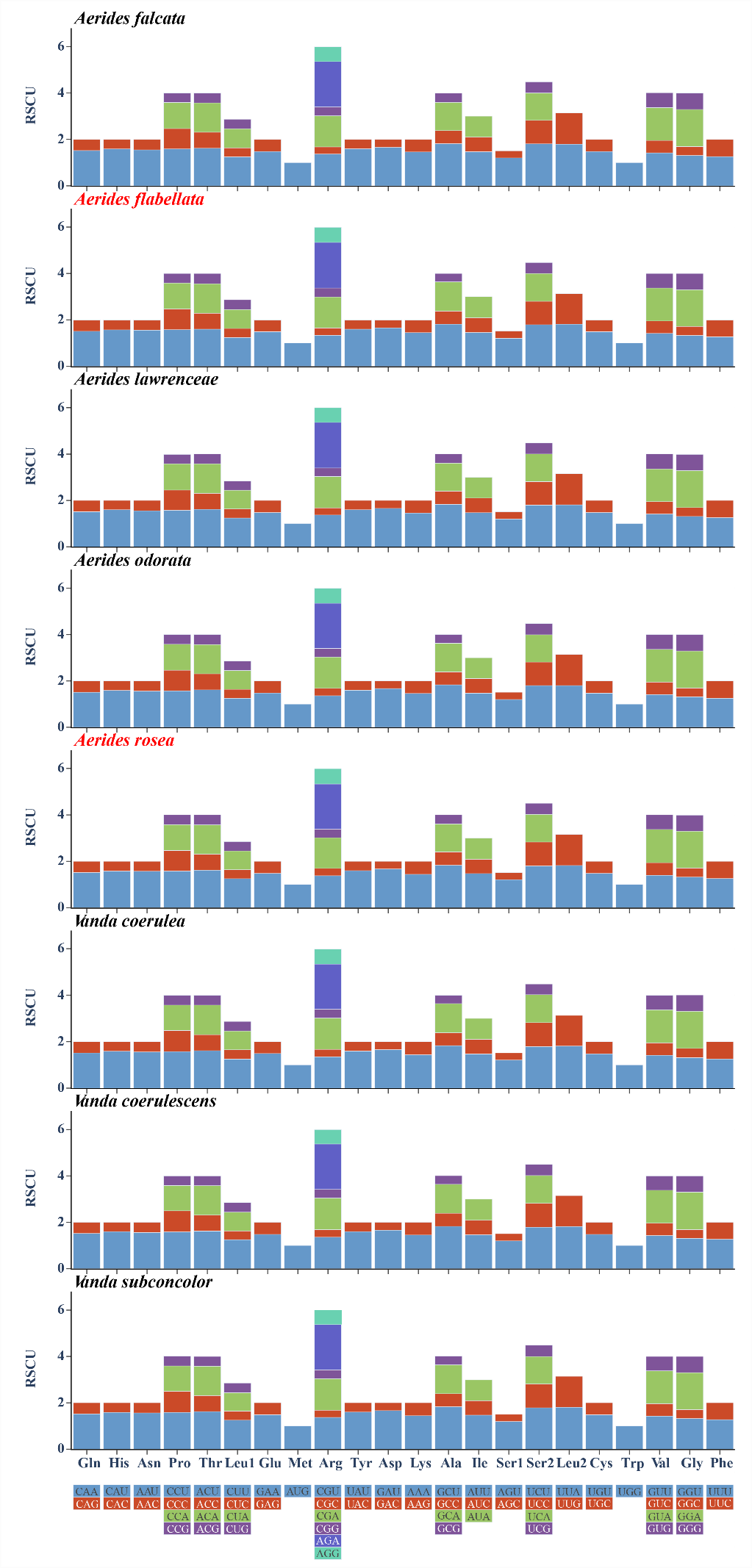


Fig. S3 Relative synonymous codon usage (RSCU) of two *Aerides* species and six species selected from “*Vanda*-*Aerides* alliance”. Amino acid families are labelled on the x-axis. The histogram above each amino acid showing codon usage. Colors in the column graph reflecting codons in the same colors shown below the figure.
