# Supplementary material for: Complete chloroplast genome structural characterization of two Aerides (Orchidaceae) species with a focus on phylogenetic position of Aerides flabellata
Source: BMC Genomics. 2024 Jun 3;25:552. doi: 10.1186/s12864-024-10458-0 (PMC11145882; doi:10.1186/s12864-024-10458-0)
Supplement: Supplementary file 2 — Supplementary Material 2. [file 12864_2024_10458_MOESM2_ESM.doc]

Table S1 Genes annotation for the chloroplast genome of the two *Aerides* species.

| **Categories of genes** | **Groups of genes** | **Name of genes** |
| --- | --- | --- |
| Genetic genes | Large subunit of ribosome (LSU) | *rpl2**(x2), rpl14, rpl16*, rpl20, rpl22, rpl23(x2), rpl32, rpl33, rpl36 |
| System | Small subunit of ribosome (SSU) | *rps2*, rps3, rps4, rps7(x2), rps8, rps11, rps12**(x2), rps14, rps15, rps16*, rps18, rps19(x2) |
|  | RNA polymerase | *rpoA*, rpoB, rpoC1*, rpoC2 |
|  | Ribosomal RNA (rRNA) | *rrn4.5*(x2), rrn5(x2), rrn16(x2), rrn23(x2) |
|  | Transfer RNA (tRNA) | *trnA-UGC*(x2), trnC-GCA, trnD-GUC, trnE-UUC, trnF-GAA, trnfM-CAU, trnG-GCC, trnG-UCC, trnH-GUG(x2), trnI-CAU(x2), trnI-GAU(x2), trnK-UUU,  trnL-CAA(x2), trnL-UAA, trnL-UAG, trnM-CAU, trnN-GUU(x2), trnP-UGG, trnQ-UUG, trnR-ACG(x2), trnR-UCU, trnS-GCU,  trnS-GGA, trnS-UGA, trnT-GGU, trnT-UGU, trnV-GAC(x2), trnV-UAC,  trnW-CCA, trnY-GUA |
|  | Translational initiation factor | *infA* |
| Photosynthetic genes | Photosystm I | *psaA*, psaB, psaC, psaI, psaJ |
|  | Photosystem II | *psbA*, psbB, psbC, psbD, psbE, psbF, psbH, psbI, psbJ, psbK, psbL, psbM, psbN, psbT, psbZ |
|  | NADH dehydrogenase | ndhC, ndhD, ndhE^1^, ndhG^2^, *ndhI*, ndhJ, ndhK |
|  | Cytochrome b/f complex | *petA*, petB*, petD*, petG, petL, petN |
|  | ATP synthase | *atpA*, atpB, atpE, atpF*, atpH, atpI |
|  | Large subunit of Rubisco | *rbcL* |
| Biosynthesis genes | Maturase | *matK* |
|  | ATP-dependent protease proteolytic subunit | *clpP*** |
|  | Cov envolpe membrane protein | *cemA* |
|  | Acetyl-CoA-carboxylase | *accD* |
|  | C-type cytchrome synthesis gene | *ccsA* |
|  | Hypothetic chloroplast reading frames | *ycf1*, ycf2(x2), ycf3**, ycf4 |

*: contains one intron; **: contains two introns; (×2): genes located in IRs; ^1^: gene in *Aerides flabellata* only; ^2^: gene in *A. rosea* only.

Table S2 The genes with introns and the lengths of exons and intron.

| **Gene** | **Location** | **Exon Ⅰ (bp)** | **Intron Ⅰ (bp)** | **Exon Ⅱ (bp)** | **Intron Ⅱ (bp)** | **Exon Ⅲ (bp)** |
| --- | --- | --- | --- | --- | --- | --- |
| *clpP*^1^ | LSC | 252 | 671 | 292 | 1000 | 149 |
| *clpP*^2^ | LSC | 252 | 677 | 292 | 1000 | 149 |
| *ycf3*^1^ | LSC | 153 | 747 | 228 | 709 | 126 |
| *ycf3*^2^ | LSC | 153 | 748 | 228 | 729 | 126 |
| *rps12* | LSC | 114 |  | 232 | 547 | 26 |
| *rps16*^1^ | LSC | 40 | 904 | 245 |  |  |
| *rps16*^2^ | LSC | 40 | 924 | 230 |  |  |
| *rpoC1*^1^ | LSC | 453 | 757 | 1608 |  |  |
| *rpoC1*^2^ | LSC | 453 | 755 | 1608 |  |  |
| *rpl2* | IR | 385 | 664 | 431 |  |  |
| *rpl16*^1^ | LSC | 9 | 1295 | 399 |  |  |
| *rpl16*^2^ | LSC | 9 | 1187 | 399 |  |  |
| *petD*^1^ | LSC | 8 | 836 | 484 |  |  |
| *petD*^2^ | LSC | 8 | 851 | 484 |  |  |
| *petB*^1^ | LSC | 6 | 745 | 642 |  |  |
| *petB*^2^ | LSC | 6 | 725 | 642 |  |  |
| *atpF*^1^ | LSC | 145 | 983 | 398 |  |  |
| *atpF*^2^ | LSC | 145 | 984 | 410 |  |  |

^1^: gene in *Aerides flabellata*; ^2^: gene in *A. rosea*.

Table S3 The details information of long repeats in the two *Aerides* species.

| **Sequence length** | **Species** | ***Aerides flabellata*** | ***Aerides rosea*** |
| --- | --- | --- | --- |
| 40+ | Complement | 0 | 0 |
|  | Forward | 0 | 8 |
|  | Palindrome | 3 | 13 |
|  | Reverse | 0 | 0 |
| 30-39 | Complement | 1 | 0 |
|  | Forward | 4 | 5 |
|  | Palindrome | 9 | 10 |
|  | Reverse | 3 | 5 |
| 20-29 | Complement | 1 | 0 |
|  | Forward | 7 | 2 |
|  | Palindrome | 13 | 5 |
|  | Reverse | 8 | 1 |
