# Supplementary material for: Complete chloroplast genome structural characterization of two Aerides (Orchidaceae) species with a focus on phylogenetic position of Aerides flabellata
Source: BMC Genomics. 2024 Jun 3;25:552. doi: 10.1186/s12864-024-10458-0 (PMC11145882; doi:10.1186/s12864-024-10458-0)
Supplement: Supplementary file 4 — Supplementary Material 4. [file 12864_2024_10458_MOESM4_ESM.doc]

Table S5 Comparison of the nucleotide variability (PI) among CDS regions of the two *Aerides* species and six species selected from “*Vanda*-*Aerides* alliance”.

| **Gene** | **Pi value** | **Region** |
| --- | --- | --- |
| *accD* | 0.00766369047619048 | LSC |
| *atpA* | 0.00494469441319835 | LSC |
| *atpB* | 0.00505773451665235 | LSC |
| *atpE* | 0.00123456790123457 | LSC |
| *atpF* | 0.00631412786108919 | LSC |
| *atpH* | 0.00203252032520325 | LSC |
| *atpI* | 0.00336021505376344 | LSC |
| *cemA* | 0.00445134575569358 | LSC |
| *clpP* | 0.00221755368814192 | LSC |
| *infA* | 0.00106837606837607 | LSC |
| *matK* | 0.00720307578284232 | LSC |
| *petA* | 0.00482124313900015 | LSC |
| *petB* | 0.0017636684303351 | LSC |
| *petD* | 0.00152439024390244 | LSC |
| *petG* | 0 | LSC |
| *petL* | 0.00260416666666667 | LSC |
| *petN* | 0 | LSC |
| *psaA* | 0.00391541436814406 | LSC |
| *psaB* | 0.0033041788143829 | LSC |
| *psaI* | 0.0045045045045045 | LSC |
| *psaJ* | 0.00185185185185185 | LSC |
| *psbA* | 0.00299300511164918 | LSC |
| *psbB* | 0.00268968098044719 | LSC |
| *psbC* | 0.00226039783001808 | LSC |
| *psbD* | 0.0025894538606403 | LSC |
| *psbE* | 0 | LSC |
| *psbF* | 0 | LSC |
| *psbH* | 0.00112612612612613 | LSC |
| *psbI* | 0.00965250965250965 | LSC |
| *psbJ* | 0.00813008130081301 | LSC |
| *psbK* | 0.00288018433179724 | LSC |
| *psbL* | 0.00488400488400488 | LSC |
| *psbM* | 0.00238095238095238 | LSC |
| *psbN* | 0 | LSC |
| *psbT* | 0.017526455026455 | LSC |
| *psbZ* | 0.00226757369614512 | LSC |
| *rbcL* | 0.00587919594067135 | LSC |
| *rpl14* | 0.00493612078977933 | LSC |
| *rpl16* | 0.00183823529411765 | LSC |
| *rpl20* | 0.00938256658595642 | LSC |
| *rpl22* | 0.00674603174603175 | LSC |
| *rpl33* | 0.0106609808102345 | LSC |
| *rpl36* | 0 | LSC |
| *rpoA* | 0.00750211327134404 | LSC |
| *rpoB* | 0.00420168067226891 | LSC |
| *rpoC1* | 0.00427170868347339 | LSC |
| *rpoC2* | 0.0069977192618702 | LSC |
| *rps2* | 0.0032650190878039 | LSC |
| *rps3* | 0.00505544683626875 | LSC |
| *rps4* | 0.00565770862800566 | LSC |
| *rps8* | 0.00604256854256854 | LSC |
| *rps11* | 0.0139602603631381 | LSC |
| *rps14* | 0.00719000471475719 | LSC |
| *rps16* | 0.00489417989417989 | LSC |
| *rps18* | 0.0130718954248366 | LSC |
| *ycf3* | 0.00260636799098338 | LSC |
| *ycf4* | 0.00186615186615187 | LSC |
| *ccsA* | 0.0116829340431825 | SSC |
| *psaC* | 0.00203252032520325 | SSC |
| *rpl32* | 0.00472085385878489 | SSC |
| *rps15* | 0.00841097308488613 | SSC |
| *ycf1* | 0.0197037220434101 | SSC |
| *rpl2* | 0.000700280112044818 | IR |
| *rpl23* | 0.00202634245187437 | IR |
| *rps7* | 0.00297619047619048 | IR |
| *rps12* | 0.0322802197802198 | IR |
| *rps19* | 0.000896057347670251 | IR |
| *ycf2* | 0.00260432976347049 | IR |
| average | 0.005048207717 |  |

Table S6 Comparison of the nucleotide variability among IGS regions of the two *Aerides* species and six species selected from “*Vanda*-*Aerides* alliance”.

| **Gene** | **Pi value** | **Region** |
| --- | --- | --- |
| *accD_psaI* | 0.0253142857142857 | LSC |
| *atpB_rbcL* | 0.0155517349171616 | LSC |
| *cemA_petA* | 0.013677811550152 | LSC |
| *clpP_psbB* | 0.0403277123767102 | LSC |
| *matK_rps16* | 0.019672131147541 | LSC |
| *ndhC_trnV-UAC* | 0.0315315315315315 | LSC |
| *petA_psbJ* | 0.0193832249881085 | LSC |
| *psaI_ycf4* | 0.0163652802893309 | LSC |
| *psaJ_rpl33* | 0.0154330963154493 | LSC |
| *psbB_psbT* | 0.0529100529100529 | LSC |
| *psbE_petL* | 0.0843253968253968 | LSC |
| *psbK_psbI* | 0.0233023702754644 | LSC |
| *rbcL_accD* | 0.0163500182681768 | LSC |
| *rpl20_rps12* | 0.0104388169340808 | LSC |
| *rps4_trnT-UGU* | 0.0133262260127932 | LSC |
| *rps16_trnQ-UUG* | 0.0226157770800628 | LSC |
| *rps18_rpl20* | 0.0147058823529412 | LSC |
| *trnF-GAA_ndhJ* | 0.0171541057367829 | LSC |
| *trnL-UAA_trnF-GAA* | 0.0179934569247546 | LSC |
| *trnP-UGG_psaJ* | 0.0295723384895359 | LSC |
| *trnQ-UUG_psbK* | 0.0143049155145929 | LSC |
| *trnT-UGU_trnL-UAA* | 0.0237430167597765 | LSC |
| *ycf4_cemA* | 0.0123773173391494 | LSC |
| *ccsA_ndhD* | 0.0320891514500537 | SSC |
| *rpl32_trnL-UAG* | 0.0241883116883117 | SSC |
| *ndhB_rps7* | 0.00311962134251291 | IR |
| *rps7_trnV-GAC* | 0.00829198224079394 | IR |
| *rrn4.5_rrn5* | 0.00505390835579515 | IR |
| *rrn5_trnR-ACG* | 0.00720551378446115 | IR |
| *rrn16_trnI-GAU* | 0.0192762535477767 | IR |
| *trnL-CAA_ndhB* | 0.00601779173207745 | IR |
| *trnR-ACG_trnN-GUU* | 0.00993967644639429 | IR |
| *trnV-GAC_rrn16* | 0 | IR |
| *ycf2_trnL-CAA* | 0.00251026928343222 | IR |
| average | 0.019649087651 |  |

Table S7 Positive selection sites were detected in the cp genome of the two *Aerides* species and six species selected from “*Vanda*-*Aerides* alliance”.

| ***Gene*** | **M8** | **Region** |
| --- | --- | --- |
| *atpA* | 306 S 0.560, 508 - 0.988* | LSC |
| *atpE* | 23 I 0.897, 44 I 0.895, 135 - 1.000** | LSC |
| *atpH* | 82 - 1.000** | LSC |
| *atpI* | 56 I 0.647, 93 P 0.594, 248 - 1.000** | LSC |
| *cemA* | 47 S 0.942, 96 V 0.617, 132 F 0.623, 134 L 0.623, 184 A 0.545, 230 - 1.000** | LSC |
| *infA* | 78 - 1.000** | LSC |
| *matK* | 246 I 0.760 | LSC |
| *petA* | 5 N 0.659, 29 R 0.720, 223 G 0.727, 291 F 0.749, 321 - 1.000** | LSC |
| *petB* | 216 - 1.000** | LSC |
| *petD* | 164 - 1.000** | LSC |
| *petG* | 38 - 1.000** | LSC |
| *petL* | 32 - 1.000** | LSC |
| *petN* | 30 - 1.000** | LSC |
| *psaA* | 13 I 0.512, 751 - 0.877 | LSC |
| *psaB* | 359 A 0.660, 411 M 0.639, 419 T 0.662, 735 - 0.991** | LSC |
| *psaI* | 4 L 0.740, 37 - 1.000** | LSC |
| *psaJ* | 45 - 1.000** | LSC |
| *psbA* | 349 P 0.660, 354 - 1.000** | LSC |
| *psbB* | 373 A 0.691, 509 - 1.000** | LSC |
| *psbD* | 354 - 1.000** | LSC |
| *psbE* | 84 - 1.000** | LSC |
| *psbF* | 40 - 1.000** | LSC |
| *psbH* | 74 - 1.000** | LSC |
| *psbI* | 37 - 1.000** | LSC |
| *psbJ* | 41 - 1.000** | LSC |
| *psbK* | 45 L 0.912, 62 - 1.000** | LSC |
| *psbL* | 39 - 0.999** | LSC |
| *psbM* | 35 - 1.000** | LSC |
| *psbN* | 44 - 1.000** | LSC |
| *psbT* | 36 - 1.000** | LSC |
| *psbZ* | 63 - 1.000** | LSC |
| *rbcL* | 486 A 0.824 | LSC |
| *rpl14* | 4 P 0.576, 123 - 1.000** | LSC |
| *rpl16* | 22 A 0.536, 136 - 1.000** | LSC |
| *rpl20* | 47 H 0.513, 51 N 0.581, 101 V 0.601, 107 F 0.527, 113 K 0.535, 118 - 1.000** | LSC |
| *rpl22* | 1 M 0.775, 2 I 0.774, 3 K 0.780, 4 N 0.781, 5 E 0.775, 6 N 0.775, 7 R 0.775, 8 E 0.775, 9 A 0.776, 10 K 0.776, 11 V 0.775, 12 L 0.781, 13 A 0.776, 14 Q 0.781, 15 N 0.775, 16 I 0.774, 17 C 0.779, 18 M 0.775, 19 S 0.779, 20 V 0.775, 21 F 0.782, 22 K 0.776, 23 A 0.776, 24 R 0.781, 25 R 0.775, 26 V 0.776, 27 I 0.775, 28 D 0.774, 29 Q 0.782, 30 I 0.775, 31 R 0.779, 32 G 0.777, 33 R 0.779, 34 S 0.779, 35 Y 0.778, 36 E 0.780, 37 E 0.775, 38 A 0.776, 39 L 0.782, 40 M 0.775, 41 I 0.774, 42 L 0.781, 43 E 0.775, 44 L 0.781, 45 M 0.775, 46 P 0.779, 47 Y 0.778, 48 R 0.780, 49 A 0.776, 50 S 0.779, 51 Y 0.778, 52 P 0.779, 53 I 0.782, 54 L 0.781, 55 K 0.982*, 56 L 0.781, 57 V 0.775, 58 Y 0.982*, 59 S 0.779, 60 A 0.776, 61 A 0.776, 62 A 0.776, 63 N 0.775, 64 A 0.776, 65 S 0.774, 66 H 0.778, 67 N 0.775, 68 M 0.775, 69 G 0.776, 70 L 0.782, 71 N 0.775, 72 E 0.775, 73 V 0.775, 74 D 0.774, 75 L 0.781, 76 F 0.778, 77 I 0.775, 78 S 0.774, 79 K 0.776, 80 A 0.776, 81 E 0.775, 82 V 0.782, 83 N 0.775, 84 R 0.775, 85 G 0.776, 86 T 0.775, 87 I 0.982*, 88 V 0.982*, 89 K 0.776, 90 K 0.780, 91 L 0.781, 92 K 0.776, 93 P 0.782, 94 R 0.782, 95 A 0.776, 96 R 0.781, 97 G 0.777, 98 R 0.779, 99 S 0.774, 100 Y 0.778, 101 L 0.782, 102 I 0.774, 103 K 0.776, 104 K 0.776, 105 T 0.782, 106 T 0.775, 107 C 0.779, 108 H 0.778, 109 I 0.774, 110 N 0.982*, 111 I 0.775, 112 F 0.778, 113 L 0.781, 114 K 0.776, 115 E 0.775, 116 K 0.776, 117 Y 0.778, 118 K 0.780, 119 I 0.775, 120 - 1.000** | LSC |
| *rpl33* | 20 R 0.539, 67 - 1.000** | LSC |
| *rpl36* | 38 - 1.000** | LSC |
| *rpoA* | 3 Q 0.635, 34 M 0.627, 44 T 0.668, 115 Y 0.671, 146 C 0.579, 166 H 0.659, 239 F 0.654, 261 K 0.548, 334 K 0.535, 337 F 0.643, 338 - 1.000** | LSC |
| *rpoC1* | 14 P 0.558, 21 R 0.569, 85 K 0.532, 181 Q 0.568, 418 I 0.891, 562 R 0.542, 581 Y 0.543, 598 H 0.890 | LSC |
| *rpoC2* | 479 G 0.818, 696 I 0.825, 923 L 0.818, 1007 N 0.809 | LSC |
| *rps3* | 19 L 0.671, 113 A 0.606, 195 H 0.728, 219 - 1.000** | LSC |
| *rps4* | 69 M 0.629, 166 I 0.965*, 190 E 0.594, 202 - 1.000** | LSC |
| *rps8* | 3 R 0.967*, 72 K 0.679, 85 R 0.660, 132 - 1.000** | LSC |
| *rps11* | 77 F 0.595, 139 - 1.000** | LSC |
| *rps14* | 22 F 0.960*, 25 E 0.568, 101 - 1.000** | LSC |
| *rps16* | 1 M 0.783, 2 V 0.783, 3 K 0.783, 4 L 0.783, 5 R 0.783, 6 L 0.783, 7 K 0.783, 8 R 0.783, 9 C 0.783, 10 G 0.783, 11 R 0.783, 12 K 0.783, 13 Q 0.783, 14 R 0.783, 15 A 0.783, 16 I 0.783, 17 Y 0.783, 18 R 0.783, 19 I 0.783, 20 V 0.783, 21 A 0.783, 22 I 0.783, 23 D 0.783, 24 V 0.783, 25 R 0.783, 26 S 0.783, 27 R 0.783, 28 R 0.783, 29 E 0.783, 30 G 0.783, 31 R 0.783, 32 D 0.783, 33 L 0.783, 34 Q 0.977*, 35 K 0.783, 36 V 0.783, 37 G 0.783, 38 F 0.977*, 39 Y 0.783, 40 D 0.783, 41 P 0.783, 42 I 0.783, 43 K 0.783, 44 N 0.783, 45 Q 0.783, 46 T 0.783, 47 Y 0.783, 48 S 0.783, 49 N 0.783, 50 V 0.783, 51 P 0.783, 52 A 0.783, 53 I 0.783, 54 L 0.783, 55 Y 0.783, 56 F 0.783, 57 L 0.783, 58 E 0.783, 59 K 0.783, 60 G 0.783, 61 A 0.783, 62 Q 0.783, 63 P 0.783, 64 T 0.783, 65 E 0.783, 66 T 0.783, 67 V 0.783, 68 Y 0.783, 69 D 0.783, 70 I 0.783, 71 L 0.783, 72 S 0.783, 73 K 0.783, 74 T 0.783, 75 E 0.783, 76 F 0.783, 77 F 0.783, 78 K 0.783, 79 E 0.783, 80 F 0.783, 81 R 0.783, 82 I 0.783, 83 S 0.783, 84 F 0.783, 85 D 0.783, 86 K 0.977*, 87 K 0.783, 88 R 0.783, 89 K 0.783 | LSC |
| *rps18* | 4 F 0.964*, 102 - 1.000** | LSC |
| *ycf3* | 128 S 0.505 | LSC |
| *ycf4* | 112 R 0.597, 185 - 1.000** | LSC |
| *ccsA* | 182 R 0.562, 184 P 0.570, 192 Y 0.937, 205 F 0.525, 322 - 1.000** | SSC |
| *psaC* | 82 - 1.000** | SSC |
| *rpl32* | 52 K 0.957*, 58 - 1.000** | SSC |
| *ycf1* | 47 A 0.835, 66 V 0.533, 98 L 0.836, 150 A 0.547, 245 T 0.883, 247 M 0.515, 253 I 0.792, 254 E 0.859, 272 F 0.538, 278 S 0.545, 306 H 0.874, 311 Y 0.515, 328 E 0.817, 344 W 0.548, 440 Y 0.522, 489 F 0.507, 561 F 0.862, 574 R 0.790, 629 E 0.852, 632 Q 0.515, 651 L 0.863, 708 L 0.834, 748 A 0.842, 760 S 0.824, 772 Y 0.532, 781 A 0.546, 796 N 0.855, 806 I 0.518, 813 S 0.508, 829 E 0.971*, 863 T 0.804, 925 L 0.542, 926 L 0.858, 1020 L 0.976*, 1041 K 0.775, 1077 R 0.796, 1085 F 0.836, 1105 S 0.543, 1109 W 0.549, 1361 N 0.784, 1383 Y 0.539, 1483 R 0.791, 1501 M 0.804, 1506 I 0.808, 1535 L 0.523, 1545 V 0.527, 1562 S 0.863, 1596 R 0.874, 1643 R 0.553, 1648 V 0.505, 1685 L 0.522 | SSC |
| *rpl2* | 16 V 0.641, 272 - 1.000** | IR |
| *rpl23* | 24 S 0.875, 94 - 1.000** | IR |
| *rps7* | 156 - 1.000** | IR |
| *rps19* | 88 N 0.671, 93 - 1.000** | IR |
| *ycf2* | 166 K 0.660, 225 D 0.647, 268 M 0.648, 418 L 0.658, 473 N 0.638, 500 W 0.681, 517 H 0.660, 561 D 0.651, 562 S 0.994**, 563 G 0.985*, 564 C 0.989*, 565 D 0.643, 566 M 0.660, 637 G 0.679, 665 K 0.660, 771 M 0.971*, 958 R 0.687, 1039 N 0.637, 1139 I 0.666, 1172 N 0.665, 1184 Y 0.662, 1505 L 0.686, 1562 D 0.972*, 1573 F 0.670, 1602 L 0.654, 1664 K 0.656, 1782 N 0.969*, 1815 K 0.661, 1896 L 0.659, 1920 Y 0.657, 2152 S 0.685, 2256 R 0.669 | IR |

*p>95%; ** p>99%

Table S8 List of 62 CDS used in the phylogenetic analysis.

| **Gene** | **Numbers** | **Gene** | **Numbers** |
| --- | --- | --- | --- |
| *accD* | 54 | *psbL* | 54 |
| *atpA* | 54 | *psbM* | 54 |
| *atpB* | 54 | *psbT* | 54 |
| *atpE* | 54 | *rbcL* | 54 |
| *atpF* | 54 | *rpl2* | 54 |
| *atpH* | 54 | *rpl14* | 54 |
| *atpI* | 54 | *rpl16* | 54 |
| *cemA* | 54 | *rpl20* | 54 |
| *infA* | 54 | *rpl22* | 54 |
| *matK* | 54 | *rpl23* | 54 |
| *petA* | 54 | *rpl32* | 54 |
| *petB* | 54 | *rpl33* | 54 |
| *petD* | 54 | *rpl36* | 54 |
| *petG* | 54 | *rpoA* | 54 |
| *petL* | 54 | *rpoB* | 54 |
| *petN* | 54 | *rpoC1* | 54 |
| *psaA* | 54 | *rpoC2* | 54 |
| *psaB* | 54 | *rps2* | 54 |
| *psaC* | 54 | *rps3* | 54 |
| *psaI* | 54 | *rps4* | 54 |
| *psaJ* | 54 | *rps7* | 54 |
| *psbA* | 54 | *rps8* | 54 |
| *psbB* | 54 | *rps11* | 54 |
| *psbC* | 54 | *rps12* | 54 |
| *psbD* | 54 | *rps14* | 54 |
| *psbE* | 54 | *rps15* | 54 |
| *psbF* | 54 | *rps16* | 54 |
| *psbH* | 54 | *rps18* | 54 |
| *psbI* | 54 | *rps19* | 54 |
| *psbJ* | 54 | *ycf1* | 54 |
| *psbK* | 54 | *ycf2* | 54 |

Table S9 List of 53 species (*Aerides rosea* repeat) used in the phylogenetic analysis.

| **Species** | **Subtribe** | **GenBank accession numbers** |
| --- | --- | --- |
| *Acampe papillosa* | Aeridinae | MN124418 |
| *Acampe rigida* | Aeridinae | MN124419 |
| *Aerides crassifolia* | Aeridinae | OR159897 |
| *Aerides falcata*** | Aeridinae | OR159896 |
| *Aerides flabellata** | Aeridinae | PP003956 |
| *Aerides lawrenceae*** | Aeridinae | OR159898 |
| *Aerides odorata*** | Aeridinae | OR159899 |
| *Aerides quinquevulnera* | Aeridinae | OR159900 |
| *Aerides rosea* | Aeridinae | OR159901 |
| *Aerides rosea** | Aeridinae | PP003955 |
| *Cleisostoma arietinum* | Aeridinae | MN124420 |
| *Cleisostoma williamsonii* | Aeridinae | MN124426 |
| *Gastrochilus calceolaris* | Aeridinae | MH719016 |
| *Gastrochilus fuscopunctatus* | Aeridinae | KX871233 |
| *Gastrochilus guangtungensis* | Aeridinae | MN124428 |
| *Gastrochilus japonicus* | Aeridinae | KX871236 |
| *Holcoglossum amesianum* | Aeridinae | MK442924 |
| *Holcoglossum flavescens* | Aeridinae | MK442925 |
| *Holcoglossum nagalandense* | Aeridinae | MK442928 |
| *Holcoglossum quasipinifolium* | Aeridinae | MK442931 |
| *Holcoglossum rupestre* | Aeridinae | NC041517 |
| *Holcoglossum singchianum* | Aeridinae | MN732560 |
| *Holcoglossum tsii* | Aeridinae | MK836106 |
| *Holcoglossum wangii* | Aeridinae | MK442935 |
| *Pelatantheria scolopendrifolia* | Aeridinae | KX871232 |
| *Phalaenopsis deliciosa* | Aeridinae | OM792977 |
| *Phalaenopsis mannii* | Aeridinae | MT822270 |
| *Phalaenopsis stobartiana* | Aeridinae | MW531729 |
| *Phalaenopsis wilsonii* | Aeridinae | OP723311 |
| *Phalaenopsis zhejiangensis* | Aeridinae | MZ326749 |
| *Renanthera citrina* | Aeridinae | MZ959043 |
| *Renanthera coccinea* | Aeridinae | OK377034 |
| *Renanthera imschootiana* | Aeridinae | OK377035 |
| *Renanthera philippinensis* | Aeridinae | OK377036 |
| *Thrixspermum amplexicaule* | Aeridinae | MW574621 |
| *Thrixspermum centipeda* | Aeridinae | MW057769 |
| *Thrixspermum japonicum* | Aeridinae | KX871234 |
| *Thrixspermum tsii* | Aeridinae | MN725094 |
| *Uncifera acuminata* | Aeridinae | MN124433 |
| *Vanda brunnea* | Aeridinae | MK442937 |
| *Vanda coerulea***** | Aeridinae | MN711649 |
| *Vanda coerulescens*** | Aeridinae | MN711650 |
| *Vanda concolor* | Aeridinae | NC048458 |
| *Vanda falcata* | Aeridinae | KT726909 |
| *Vanda subconcolor*** | Aeridinae | MT180955 |
| *Vandopsis gigantea* | Aeridinae | MN124403 |
| *Vandopsis lissochiloides* | Aeridinae | MN124443 |
| *Vandopsis undulata* | Aeridinae | MN124402 |
| **Outgroup** |  |  |
| *Polystachya adansoniae* | Polystachyinae | OK930072 |
| *Polystachya bennettiana* | Polystachyinae | OK930074 |
| *Polystachya concreta* | Polystachyinae | OK930076 |
| *Polystachya dendrobiiflora* | Polystachyinae | OK930071 |
| *Polystachya modesta* | Polystachyinae | OK930075 |
| *Polystachya tenuissima* | Polystachyinae | OK930073 |

*: Sequences sequenced in this study. **: Sequences used for comparative analysis.
